# A meta-analysis of the impact of technology related factors on students’ academic performance

**DOI:** 10.3389/fpsyg.2025.1524645

**Published:** 2025-02-21

**Authors:** Metin Kuş

**Affiliations:** ^1^Physical Education and Sports Department, Faculty of Sport Sciences, Hitit University, Çorum, Türkiye; ^2^Distance Education, Application and Research Center, Hitit University, Çorum, Türkiye

**Keywords:** academic performance, meta-analysis, smartphone addiction, social media use, videogames

## Abstract

**Introduction:**

The relationship between students’ smartphone addiction, social media use, video games play, and their academic performance has been widely studied, yet the existing literature presents inconsistent findings. This meta-analysis synthesizes current research to provide a comprehensive examination of the impact of these technologies on academic achievement.

**Methods:**

A total of 63 studies (yielding 64 effect sizes) were included, encompassing a sample of 124,166 students from 28 countries. The meta-analysis utilized correlation coefficients and sample sizes, reporting results based on the random effects model. Key statistics such as the Fisher’s *Z* value, confidence intervals, and heterogeneity (*Q*) test results were considered, and publication bias was assessed using Begg and Mazumdar’s rank correlation test, with the Kendall Tau coefficient determining bias significance.

**Results and discussion:**

The meta-analysis revealed a small but statistically significant negative association between smartphone use, social media use, video game playing, and students’ academic performance [*Q*(64) = 2501.93, *p* < 0.001, *d* = −0.085]. It is concluded that increased use of these technologies was associated with poorer academic outcomes, potentially impacting key cognitive skills essential for academic success. The implications for educational psychology research and future research directions are discussed.

## 1 Introduction

The rapid proliferation of digital technologies has significantly altered the ways students engage with academic work. While smartphones, social media, and video games offer potential educational benefits, concerns have emerged about their possible detrimental effects on students’ academic performance. Research on this topic has yielded mixed findings, with some studies indicating negative effects on learning outcomes, while others suggest potential advantages under certain conditions. Despite extensive research investigating the relationships between smartphone addiction (SA), social media use (SMU), video games (VGs), and academic performance (AP), findings remain inconsistent and, at times, controversial. Most studies suggest a negative relationship between these digital behaviors and academic outcomes (Kim et al., 2019; Yang et al., 2022). However, a smaller body of research has reported either no significant impact or a positive association between SA, SMU, VGs, and AP (Sert et al., 2019; Van Den Eijnden et al., 2018). These conflicting findings may stem from variations in the purposes for which smartphones, social media, and video games are used. Smartphones have become indispensable due to their multifunctionality and portability, contributing to increased dependency. Among younger generations, smartphones are commonly used for watching sports, gaming, online banking, communication, social media engagement, and even completing homework (Nayak, 2018).

Despite the volume of research, there is no clear consensus on the direction and magnitude of the effects of SA, SMU, and VGs on AP. While most studies point to a negative association, the effect sizes reported vary widely. This inconsistency underscores the need for a meta-analytic approach to estimate the true overall impact of SA, SMU, and VGs on academic outcomes. Given the widespread prevalence of these behaviors among students, it is critical to systematically examine their relationships with academic achievement. To date, no published meta-analysis has comprehensively addressed the link between SA, SMU, VGs, and student academic performance. The present study seeks to fill this gap in the literature, providing valuable insights for scholars, educators, and policymakers. By exploring these relationships, this research aims to inform strategies that can maximize the benefits of SA, SMU, and VGs while minimizing their potential adverse effects on academic achievement.

## 2 Theoretical background

Previous studies on smartphone addiction (SA), social media use (SMU), and video game use (VGs) are informed by several theoretical frameworks. For example, Cognitive Load Theory (Sweller, 1994), posits that excessive engagement with these technologies can hinder learning by overloading working memory. Frequent notifications, multitasking, and prolonged screen time can deplete cognitive resources, leading to decreased attention and comprehension, ultimately impacting academic performance. Similarly, Self-Regulation Theory (Zimmerman, 2000), emphasizes the potential for digital distractions to impair students’ ability to manage their time and academic tasks effectively. Students with weak self-regulatory skills may be more susceptible to distractions from smartphones, social media, and video games, resulting in procrastination and reduced study time. Conversely, students who effectively regulate their digital habits may use these tools for productive academic engagement. In this regard, Social Learning Theory (Bandura, 1986), offers a contrasting perspective, suggesting that social media can facilitate knowledge sharing, and video games can enhance cognitive skills such as problem-solving and strategic thinking, potentially benefiting academic outcomes. This theory posits that digital platforms can be valuable learning and collaboration tools. Social media can foster peer learning, discussions, and access to diverse educational resources, while video games may improve cognitive functions like problem-solving, strategic thinking, and hand-eye coordination.

This study integrates these theoretical perspectives to provide a more nuanced understanding of the complex relationship between SA, SMU, VGU, and AP. Cognitive Load Theory explains the potential negative impact of excessive technology use on learning efficiency, while Self-Regulation Theory highlights the importance of students’ ability to manage their digital behavior. Social Learning Theory offers a counterpoint, suggesting that, under certain conditions, social media and video games can positively contribute to academic engagement. By synthesizing findings from previous research, this meta-analysis aims to clarify these complex interactions and provide insights into optimizing technology use for academic success. Yet, in the first place, this requires a clear conceptualization of SA, SMU, VGU, and AP.

### 2.1 Conceptualization of smartphone addiction, social media use, video games play

The widespread availability of smartphones has facilitated instant access to information, entertainment, and remote communication, profoundly shaping modern social interactions and media consumption patterns (O’Dea, 2023; Scott et al., 2016). However, this ubiquitous access has also raised concerns about smartphone addiction (SA). SA is generally defined as the compulsive and uncontrollable overuse of smartphones, which can result in various negative outcomes, including withdrawal symptoms, diminished academic performance, strained social relationships, and physical health issues, all of which can impair an individual’s daily functioning (Kuss and Griffiths, 2017). In the literature, several terms - such as “problematic mobile or smartphone use” (Kim et al., 2015), “smartphone addiction” (Jeong, 2016; Kim et al., 2017), “smartphone dependence” (Lee et al., 2016; Yoo et al., 2020), and “smartphone overuse” (Kim et al., 2019; Hwang et al., 2012), are used interchangeably to describe this phenomenon, which is characterized by an individual’s inability to regulate their smartphone use, leading to adverse consequences. Among these consequences, impairments in attention and learning have been identified as significant contributors to decreased academic performance, particularly within educational settings (Dontre, 2021; Yang et al., 2018).

The emergence of social media has significantly reshaped how individuals communicate and share information, exerting a profound impact on modern society and education. The younger generation has played a central role in this digital transformation, seamlessly integrating various social media platforms into their daily routines (Lim et al., 2021; Mansour et al., 2020). As students engage in academic activities, they encounter the pervasive influence of social media, which presents both advantages and disadvantages (Alenezi and Brinthaupt, 2022; Lim et al., 2021; Shen, 2019). Social media use (SMU), also referred to as problematic social media use, social media disorder, or social media addiction (Sun and Zhang, 2020), has not yet been officially recognized as a behavioral addiction. However, it is generally understood as the inability to control one’s social media usage, leading to impairments in various aspects of daily functioning, particularly academic performance (Andreassen and Pallesen, 2014; Boer et al., 2021). SMU has become a widespread concern among adolescents and young adults (Swain and Pati, 2021; Tezci and İçen, 2017), prompting extensive research efforts aimed at understanding the scope of the problem (Cheng et al., 2021). The concept of SMU has been defined and assessed in various ways across the literature (Sun and Zhang, 2020).

In line with the rise of social media, advancements in digital technology have also transformed leisure activities, with video games (VGs) emerging as a prominent form of global entertainment. VGs are now played by a vast and diverse audience, including a majority of young people and adolescents (Gilbert, 2023). Recent statistics indicate that the global gaming population has reached approximately 2.7 billion active participants (Statista, 2024). This widespread engagement raises important questions about the potential effects of video game play on individuals’ lives and future prospects. Although some researchers use the terms “excessive gaming” and “problematic gaming” interchangeably, these terms refer to distinct phenomena with differing outcomes (Griffiths, 2010). Excessive gaming refers to spending extensive time playing VGs (Borgonovi, 2016; Osuagwu, 2015), which may not necessarily result in negative consequences (Griffiths, 2010). Conversely, problematic gaming, often referred to as gaming addiction or gaming disorder, is characterized by compulsive and persistent gaming behavior that leads to significant disruptions in personal, social, and academic functioning (World Health Organisation, 2020). Problematic gaming has been increasingly conceptualized within the framework of behavioral addiction (Dowling, 2014; Pontes et al., 2014).

While concerns about the potential harms of gaming persist, research has also highlighted numerous positive outcomes associated with VGs (Bavelier and Green, 2019; Cai et al., 2022). VGs have been effectively integrated into educational settings as tools to support learning, helping students reinforce academic content in subjects such as mathematics and science (Hussein et al., 2022). Additionally, gaming has been found to enhance various cognitive skills, including attention, critical thinking, problem-solving, and decision-making (Nuyens et al., 2019; Reynaldo et al., 2021). However, despite these potential benefits, excessive gaming remains a cause for concern, as it has been linked to harmful effects in certain contexts (Borgonovi, 2016).

### 2.2 Review of the research inquiring the impact of smartphone addiction, social media use, video games on academic performance

Smartphone addiction (SA), characterized by excessive and compulsive smartphone use, has been associated with a range of negative outcomes, including disruptions to daily activities and adverse impacts on academic performance (Park and Lee, 2012). A substantial body of research has examined the relationship between problematic smartphone use and academic achievement, consistently reporting a negative association, albeit with varying degrees of severity (Alosaimi et al., 2016; Alotaibi et al., 2022; Arora et al., 2018; Asante and Hiadzi, 2018; Chen and Ji, 2015; Durak, 2018; Felisoni and Godoi, 2018; Han, 2022; Kim et al., 2019; Li et al., 2015; Longnecker, 2017; Mendoza et al., 2018; Olufadi, 2015; Rozgonjuk et al., 2018; Samaha and Hawi, 2016; Spiratos, 2021; Uzun and Kilis, 2019; Zhou et al., 2022). It is evident that excessive smartphone use in educational settings can impair students’ academic performance, resulting in lower grade point averages (GPA) (Lepp et al., 2014).

On the other hand, in the literature several studies purported either positive or no significant relationship between SA and AP (Bai et al., 2020; Bergdahl et al., 2020; Bun Lee, 2015; Domoff et al., 2020; Dos, 2014; Eoh et al., 2022; Fernández-Andújar et al., 2022; Lau, 2017; Lepp et al., 2015; Lin and Chiang, 2017; Przepiorka et al., 2021; Rashid and Asghar, 2016; Rosen et al., 2018; Sert et al., 2019; Winskel et al., 2019). The potential positive impact of SA on academic performance may be explained by shifting social norms, where high levels of smartphone use have become more accepted. Furthermore, smartphones, when used as educational tools, can enhance the learning process. For example, when students are permitted to use smartphones for academic purposes in class, such usage may facilitate improved learning outcomes and contribute positively to academic performance (Tessier, 2013). These divergent findings highlight the complex and multifaceted nature of SA and its impact on academic achievement, making it a subject of ongoing debate in the scholarly community.

*Hypothesis 1*: There is an association between smartphone addiction (SA) and academic performance (AP).

SMU presents both advantages and disadvantages for students’ academic pursuits (Alenezi and Brinthaupt, 2022; Lim et al., 2021; Shen, 2019). Social media platforms enable users to share personal experiences, connect with peers, and access information in ways that were once unimaginable. While these platforms offer valuable tools for enhancing learning, they can also serve as significant distractions, potentially undermining academic performance (Alenezi and Brinthaupt, 2022). The educational benefits of social media engagement are evident, but concerns have been raised regarding its potential detrimental effects on academic performance (Lim et al., 2021; Shen, 2019). Social media’s ability to rapidly disseminate information grants students access to a wealth of resources, thereby enriching their educational experiences (Lim et al., 2021; Mansour et al., 2020). However, the multitasking nature typical of SMU often leads to cognitive overload and fragmented attention (Karpinski et al., 2013), which may, in turn, hinder academic achievement. The constant influx of notifications, updates, and content poses a challenge for students striving to maintain focus on their studies (Karpinski et al., 2013). Given these dynamics, the relationship between SMU and academic performance has become a critical area of academic inquiry. Numerous studies have explored this connection, frequently identifying a negative association between SMU and academic outcomes (Alotaibi et al., 2022; Bhandarkar et al., 2021; Gazibara et al., 2020; Homaid, 2022; Molla-Esparza et al., 2020; Pedersen et al., 2018; Qahri-Saremi and Turel, 2016; Sampasa-Kanyinga et al., 2019; Shafiq and Parveen, 2023; Shen, 2019; Tsai and Liu, 2015; Tsitsika et al., 2014; Wakefield and Frawley, 2020; Van Den Eijnden et al., 2018). Nevertheless, some research has reported either a positive or no significant relationship between SMU and academic performance (Bardakcı, 2019; Çimen and Yılmaz, 2017; Dubuc et al., 2020; Lim et al., 2021; Kalam et al., 2023; Mansour et al., 2020; Masalimova et al., 2023; Tang and Patrick, 2018; Tomé-Fernández et al., 2020). These inconsistencies in the literature suggest a complex and multifaceted relationship between SMU and AP.

*Hypothesis 2*: There is an association between social media use (SMU) and academic performance (AP).

A growing body of research has highlighted the diverse benefits of video games (VGs) in various contexts, including education (Bavelier and Green, 2019; Cai et al., 2022). VGs have been effectively integrated into educational settings to provide supplemental learning experiences that support and enhance traditional educational methods (Hussein et al., 2019; Hussein et al., 2022). Furthermore, video games have been shown to improve cognitive abilities such as attention, critical thinking, problem-solving, and decision-making (Nuyens et al., 2019; Reynaldo et al., 2021). Gameplay often requires players to maintain focus, assess situations, and develop strategies to overcome challenges and progress through levels (Borgonovi, 2016).

While these benefits are evident, excessive video game use can have detrimental effects. Excessive gaming may hinder academic success, whereas moderate gaming has been linked to potential improvements in students’ academic performance (Bavelier and Green, 2019). On the contrary, excessive gaming is associated with lower academic grades (Ferguson, 2015), and a range of other negative outcomes, including reduced cognitive abilities (Nuyens et al., 2017), vision problems (Mylona et al., 2020), musculoskeletal pain (Kumar et al., 2023), sleep disturbances (Alimoradi et al., 2019; Lam, 2014), and poor nutrition (Ayenigbara, 2018). One of the primary concerns related to problematic gaming is its negative effect on students’ academic performance (Kumar et al., 2021). Numerous studies have investigated the relationship between problematic gaming and academic performance, consistently finding a negative correlation between the two (Brunborg et al., 2014; Ekşi et al., 2020; Ferguson, 2015; Hawi et al., 2018; Jaafar et al., 2021; Karnadi and Pangestu, 2021; Khan et al., 2014; Polat and Topal, 2022; Rehbein et al., 2015; Sahin et al., 2016; Samaha and Hawi, 2020; Schmitt and Livingston, 2015; Suryawanshi et al., 2021; Toker and Baturay, 2016; Yan and Yang, 2017; Zahra et al., 2020; Zhang et al., 2019; Zorbaz et al., 2015).However, some research has reported either a positive or no significant relationship between problematic gaming and academic outcomes (Al Asqah et al., 2020; Ciris et al., 2022; ELNahas et al., 2018; Van Den Eijnden et al., 2018; Nuyens et al., 2019; Reynaldo et al., 2021).These mixed findings underscore the complex nature of video gaming and its varied impacts on academic achievement.

*Hypothesis 3*: There is an association between video game use (VG) and academic performance (AP).

The dependent variable in this study is academic performance (AP), which is defined as the overall achievement demonstrated by students throughout their educational endeavors, spanning from primary to tertiary levels (Kumar et al., 2021). AP is a key indicator of students’ knowledge acquisition, comprehension, and the ability to apply learned material (Kumar et al., 2021). Previous research has employed a variety of methods to assess AP, including standardized tests, teacher evaluations, and classroom observations (Borgonovi, 2016; Kuh et al., 2014). Additionally, some studies have utilized self-evaluation rating scales, where students assess their own academic performance (Benjet et al., 2023), while others have relied on more objective measures such as academic grades or grade point averages (GPAs) as indicators of cumulative achievement (York et al., 2015). Generally, higher academic grades or GPAs reflect stronger academic performance.

Several studies have examined the combined effects of SA, SMU, and VGs on AP, frequently identifying a negative correlation (Alotaibi et al., 2022; Bhandarkar et al., 2021; Gazibara et al., 2020; Kim et al., 2019; Samaha and Hawi, 2020; Suryawanshi et al., 2021). However, other research has reported either positive or non-significant correlations between these factors and AP (Al Asqah et al., 2020; Bai et al., 2020; Fernández-Andújar et al., 2022; Kalam et al., 2023; Masalimova et al., 2023; Reynaldo et al., 2021). These contracting findings underscore the need for further investigation into the combined influence of SA, SMU, and VGs use on academic outcomes.

Hypothesis 4: There is a combined association among smartphone addiction (SA), social media use (SMU), video game (VGs) use, and academic performance (AP).

In light of the aforementioned considerations, and the profound influence of the digital age, it is essential to carefully examine the relationships between SA, SMU, VGs, and AP. Despite the growing body of literature, no consensus exists on the extent to which SA, SMU, and VGs impact academic performance. The present study seeks to address this gap through a systematic review and meta-analysis, synthesizing findings from 63 studies across multiple educational contexts. Specifically, this study aims to investigate potential relationships between these variables. Thus, the following research questions were formulated:

**RQ1:** How do past research demonstrate the relationships between SA, SMU, VGs, and AP?

**RQ2:** What factors moderate the relationship between SA, SMU, VGs, and AP as demonstrated by past research?

By addressing these research questions, this study contributes to a more nuanced understanding of how digital behaviors shape academic outcomes, offering practical insights for optimizing technology use in educational settings.

## 3 Methods

The present study employed a meta-analytical approach to analyze the overall impact of technology related factors including smartphone addiction, social media use, and video games play on students’ academic performance. This meta-analysis utilized a robust review protocol which is consistent with the PRISMA protocol (Kitchenham and Charters, 2007; Moher et al., 2015; Page et al., 2021).

### 3.1 Eligibility criteria

Informed by a thorough review of the relevant literature, specific eligibility criteria were established to examine the association between SA, SMU, VGs, and AP. For the purpose of minimizing publication bias, the aim was to retrieve data from both published and unpublished studies.

Inclusion Criteria: Studies were considered eligible if they met the following conditions:

(i) The research focused on the impact of SA, SMU, and VGs on AP,
(ii) Academic performance was reported in terms of GPA, standardized test scores, or self-reported measures,
(iii) Sufficient and appropriate data were provided to facilitate the calculation of effect sizes,
(iv) The studies were publicly accessible either online or through library archives,
(v) The studies were published in English.

Exclusion criteria. To enhance the replicability of this meta-analysis, studies were excluded if they met any of the following conditions:

(i) The study was outside the scope of the research focus or assessed a multidimensional or complex phenomenon,
(ii) The results pertained to affective variables such as attitude or motivation,
(iii) The study did not report sufficient data to calculate effect sizes,
(iv) The sample size was fewer than 30 participants (Lin, 2018).

### 3.2 Data sources and search strategies

The following two databases were searched for potentially eligible studies: Web of Science, and Google Scholar. To conduct a comprehensive and systematic search, the following keywords were used: “smartphone addiction” OR “problematic smartphone use” OR “excessive smartphone use,” “social media addiction” OR “problematic social media use” OR “excessive social media use,” “videogame addiction” OR “problematic videogame use” OR “excessive videogame use,” and “academic performance” OR “academic achievement” OR “GPA.” A thorough literature search was carried out by two independent researchers in September 2023, across these major databases. Given the fast-evolving nature of social media research, only literature from the past decade (2014–2023) was included. The researcher independently selected studies through a sequential review of (a) their titles/abstracts and (b) their full texts (Freelon, 2013). In cases where duplicate data were identified, only data from peer-reviewed publications were used.

### 3.3 Results of the search strategy

**Figure 1:**
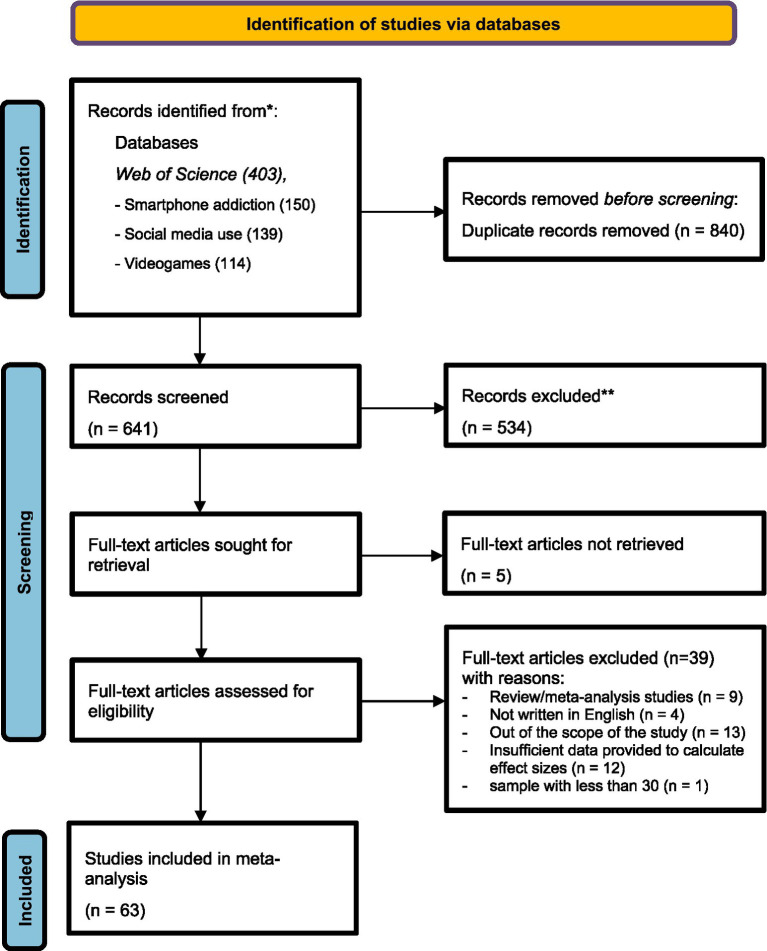
PRISMA 2020 flow diagram for meta-analysis (Page et al., 2021).

A total of 1,481 articles were initially gathered through the search process and entered into a comprehensive coding form. This meta-analysis employed a two-phase screening approach to apply the inclusion and exclusion criteria. In the first phase, both the first and second authors independently reviewed the titles and keywords of the articles to assess their relevance and ensure the inclusion criteria were strictly followed. During this phase, 840 duplicate articles were removed. Additionally, studies that did not meet the selection criteria were excluded, (n:534) leaving 107 articles after the first phase. In the second phase, the full texts of the remaining articles were obtained and carefully reviewed by two researchers. A coding sheet was developed for this stage, and both researchers independently evaluated the full texts to determine their suitability based on the inclusion criteria. As a result, only 63 articles met the criteria and were included in the final analysis (Figure 1). The coding variables included 13 categorical moderators: (a) authors’ names and publication year, (b) study title, (c) country, (d) sampling group, (e) gender, (f) age group, (g) educational level, (h) empirical approach, (i) research design, (j) academic performance indicators, (k) smartphone addiction constructs, (l) social media use constructs, and (m) video game constructs. These moderator variables were determined *a priori* and incorporated study characteristics into the coding forms and this process was guided by hypothesis and research questions of the study.

### 3.4 Data analysis procedure

After coding all the data and ensuring they met the inclusion criteria for analysis, the prepared dataset was transferred to the JAMOVI 2.5 software package for final verification. Appropriate analysis methods were then selected. Using the MAJOR add-on within JAMOVI, the correlation coefficient was calculated based on the author names, sample sizes (*n* values), and *r* values from the studies. A random-effects model was applied to interpret the findings (Jeong, 2016; Kim et al., 2017). This model assumes that errors arise not only from sampling procedures but also from additional between-study variance (Jeong, 2016; Yoo et al., 2020). In analyses using this method, effect sizes are adjusted by the inverse of the variance’s weight, accounting for both sampling error and between-study error (Yoo et al., 2020). Effect sizes in the study were calculated following Cohen’s (1992), guidelines (Lee et al., 2016). The *I*-square (*I*^2^) statistic was used to estimate the degree of confidence interval overlap and is interpreted as indicating low (25%), moderate (50%), or high (75%) levels of total variance attributable to covariates (Kim et al., 2019). A high *I*^2^ value suggests significant heterogeneity, supporting the use of a random-effects model for the meta-analysis (Field and Gillett, 2010; Hwang et al., 2012).

### 3.5 Publication bias

Meta-analyses are often susceptible to publication bias, where studies with significant results are more likely to be published, potentially skewing the overall effect size when synthesizing results from multiple studies (Dontre, 2021; Kim et al., 2017; Yang et al., 2018). To address this issue, we assessed the symmetry of the effect distribution by visually inspecting funnel plots and performing Begg and Mazumdar's (1994) regression tests (Alenezi and Brinthaupt, 2022; Lim et al., 2021; Mansour et al., 2020). Additionally, we used trim-and-fill analyses (Shen, 2019), to estimate the number of potentially missing studies and evaluate their impact on the overall meta-analytic effect. Each study provided details on the number of participants, effect size (Pearson’s *r*), confidence intervals (lower and upper bounds), relative weight, residual values, and the summary effect size if excluded from the analysis. Descriptive statistics were visualized using Microsoft Excel. For moderator analyses, appropriate criteria were applied, and the relevant analyses were incorporated accordingly.

### 3.6 Evaluation criteria for quality assessment of related articles

To rigorously assess the methodological quality of the studies included in this meta-analysis, Guidelines of Kitchenham and Charters (2007) were used. While most quality checklists in existing academic literature adhere to a combination of established guidelines, this study proposed a set of questions derived from widely used checklists and guidelines. These questions were designed to evaluate the design, conduct, analysis, and conclusions of each study included in the meta-analysis. The Evaluation Criteria (EC) presented below:

EC1: The aim of the study was clearly defined.

EC2: The context in which the study was conducted adequately described.

EC3: The research design was appropriate for addressing the study aims.

EC4: The characteristics of participants were clearly defined.

EC5: The data collection methods of the study were thoroughly described.

EC6: The study has received at least 10 citations.

EC7: Study provided a detailed description and justification of the data analysis procedures.

EC8: Results of the study were clearly presented.

EC9: Discussion and conclusion clearly compare the findings of the study with existing literature.

EC10: The study contributes to existing literature.

The scoring procedure assigned a value of 1 for “Yes” and 0 for “No,” allowing studies to score between 0 and 10 points. Papers with a score greater than 8 (>8) were selected for inclusion in this meta-analysis. The results of the quality assessment are presented in Table 1.

**Table 1:** Result of quality assessment.

Study	Publication titles	Number of citations	Quality score
Smartphone use and academic performance			
Alosaimi et al. (2016)	Saudi Medica Journal,	306	8
Chen and Ji (2015)	Computers in Human Behavior	34	10
Felisoni and Godoi (2018)	Computers and Education	150	9
Hawi and Samaha (2016)	Computers and Education	383	9
Jankovic et al. (2016)	Computers in Human Behavior	112	10
Kim et al. (2019)	Computers and Education	70	8
Lepp et al. (2014)	Computers in Human Behavior	973	10
Lepp et al. (2015)	SAGE Open	421	10
Li et al. (2015)	Computers in Human Behavior	194	10
Lin and Chiang (2017)	International Journal of Mobile Communications	38	8
Nayak (2018)	Computers and Education	202	9
Olufadi (2015)	Computers and Education	12	8
Rashid and Asghar (2016)	Computers in Human Behavior	588	10
Rosen et al. (2018)	Psicologia Educativa	39	8
Samaha and Hawi (2016)	Computers in Human Behavior	1,251	10
Sert et al. (2019)	Fatigue Biomedicine Health and Behavior	47	8
Uzun and Kilis (2019)	Computers in Human Behavior	69	9
Wentworth and Middleton (2014)	Computers and Education	157	9
Winskel et al. (2019)	Heliyon	19	9
Winskel et al. (2019)	Heliyon	19	9
Han and Yi (2019)	Technology in Society	13	8
Bai et al. (2020)	Children and Youth Services Review	45	8
Coskun and Karayagız Muslu (2019)	Community Mental Health Journal	13	8
Domoff et al. (2020)	Human Behavior and Emerging Technologies	44	8
Eoh et al. (2022)	Applied Research in Quality of Life	13	8
Przepiorka et al. (2021)	Computers and Education	13	8
Przepiorka et al. (2021)	Computers and Education	13	8
Wu and Siu (2020)	Frontiers in Psychology	16	9
Zhou et al. (2022)	Computers in Human Behavior	20	9
Social media use and academic performance			
Shafiq and Parveen (2023)	International Journal of Educational Development	19	8
Homaid (2022)	Addictive Behaviors	28	9
Dubuc et al. (2020)	International Journal of Environmental Research and Public Health	36	8
Tomé-Fernández et al. (2020)	International Journal of Environmental Research and Public Health	13	8
Bergdahl et al. (2020)	Computers and Education	144	9
Wakefield and Frawley (2020)	Computers and Education	48	8
Alotaibi (2019)	IEEE Access	50	8
Bardakcı (2019)	International Review of Research in Open and Distributed Learning	25	8
Sampasa-Kanyinga et al. (2019)	The Journal of Primary Prevention	87	9
Van Den Eijnden et al. (2018)	Journal of Behavioral Addiction	187	9
Tang and Patrick (2018)	Computers in Human Behavior	29	9
Arora et al. (2018)	Journal of Adolescent Health	13	9
Çimen and Yılmaz (2017)	Education and Science	8	8
Yan and Yang (2017)	International Journal of Environmental Research and Public Health	110	9
Qahri-Saremi and Turel (2016)	Computers and Education	48	8
Tsai and Liu (2015)	Social Psychology of Education	25	8
Tsitsika et al. (2014)	Journal of Adolescent Health,	213	9
Khan et al. (2014)	Computers and Education	73	8
Videogames use and academic performance			
Brunborg et al. (2014)	Journal of Behavioral Addictions	407	9
Schmitt and Livingston (2015)	Cyberpsychology Behavior and Social Networking	61	8
Zorbaz et al. (2015)	Educational Sciences: Theory and Practice	43	8
Sahin et al. (2016)	Educational Psychology	37	8
Hawi et al. (2018)	Journal of Behavioral Addictions	195	9
ELNahas et al. (2018)	Addictive Disorders and Their Treatment	20	8
Shi et al. (2019)	International Journal of Mental Health and Addiction	13	8
Samaha and Hawi (2020)	International Journal of Cyber Behavior Psychology and Learning	4	8
Zahra et al. (2020)	Pakistan Journal of Psychological Research	8	8
Ekşi et al. (2020)	Addicta: The Turkish Journal on Addictions	0	8
Jaafar et al. (2021)	Malaysian Journal of Medical and Health Sciences,	9	8
Karnadi and Pangestu (2021)	Journal Kajian Bimbingan Dan Konseling	2	8
Suryawanshi et al., 2021	JMIR Medical Education	7	8
Ciris et al. (2022)	Journal of Education in Science Environment and Health	7	8
Polat and Topal (2022)	Journal of Educational Technology and Online Learning	4	8
Yang et al. (2022)	International Journal of Mental Health and Addiction	7	8
Total Research: 63 Total Sample Size: 124.166			

## 4 Results

### 4.1 Description of studies

A total of 1,481 initial outputs were identified, and after the study selection process, 63 studies with 64 effect sizes (*N* = 124,166) published between 2014 and 2023 were included in the meta-analysis. The main characteristics of these studies are summarized in Table 1. All 63 studies were published in peer-reviewed journals. Academic achievement was measured using grade point average (GPA), self-reported grades, or standardized test scores. Among the retrieved studies, Turkey and the USA emerged as the leading contributors, with 10 studies conducted in Turkey and 9 in the USA. The meta-analysis included samples of students across primary, secondary, and tertiary education, with participants’ ages ranging from 7 to 27 years.

The characteristics and results of the studies are presented in Table 2. According to the analysis results, the highest positive correlation in “Smartphone Use and Academic Performance” (*r* = 0.17) was reported by Domoff et al. (2020), while the highest negative correlation (*r* = −0.38) was reported by Kim et al. (2019). The median of the observed correlations was (*r* = −0.143). Regarding the variable “Social Media Use and Academic Performance,” the lowest negative relationship (*r* = −0.407) was reported by Tsai and Liu (2015), whereas the highest positive relationship (*r* = 0.8) was reported by Bardakcı (2019). The median of the studies was (*r* = −0.0485). For the variable “Videogame Use and Academic Performance,” the lowest negative relationship (*r* = −0.271) was reported by Zorbaz et al. (2015), while the only positive relationship (*r* = 0.108) was reported by Ciris et al. (2022). The median of the observed studies was (*r* = −0.13).

**Table 2:** Characteristic of studies.

Authors (Year)	*N*	*r*	Gender	Age group	Grade level	Empirical approach	Research design	Academic performance indicator	Smartphone addiction construct
Smartphone use and academic performance									
Alosaimi et al. (2016)	2,367	−0.311	Both	20–24	TE	LRA	OS	AAS	Total phone use (hours/day).
Chen and Ji (2015)	506	−0.09	Both	18–20	TE	LRA	PPS	AGPA	Total personal electronic device use (minutes/day).
Felisoni and Godoi (2018)	43	−0.301	Both	18–19	TE	LRA	PPS	GPA	Total phone use (minutes/day).
Hawi and Samaha (2016)	293	−0.2	Both	20–24	TE	LRA	OS	AGPA	Smartphone addiction scale
Jankovic et al. (2016)	485	−0.111	Both	18–19	TE	LRA	PPS	CAS	Total phone use (hours/day).
Kim et al. (2019)	84	−0.38	Both	18–20	TE	LRA	PPS	SGPA	Total phone use in class
Lepp et al. (2014)	536	−0.203	Both	20–24	TE	PA	PPS	AGPA	-
Lepp et al. (2015)	536	−0.234	Both	20–24	TE	LRA	PPS	AGPA	Total phone use (minutes/day).
Li et al. (2015)	516	−0.173	Both	18–29	TE	PA	OS	SGPA	Total phone use (minutes/day).
Lin and Chiang (2017)	438	−0.079	Both	20–24	TE	PA	OS	SGPA	Smartphone dependency sympton scale
Nayak (2018)	429	−0.276	Both	20–24	TE	CA	PPS	APS	Total phone use (minutes/day).
Olufadi (2015)	286	−0.06	Both	20–24	TE	LRA	PPS	SGPA	Mobile phone use behaviors scale
Rashid and Asghar (2016)	761	−0.01	Both	18–22	TE	PA	OS	SGPA	Media and technology use and attitude scale
Rosen et al. (2018)	216	−0.13	Both	18–26	TE	CA	PPS	SSCG	Total phone use (minutes/day).
Hawi and Samaha (2016)	293	-0.143	Both	17–26	TE	LRA	OS	AGPA	Smartphone addiction scale
Sert et al. (2019)	743	0.047	Both	18–24	TE	CA	PPS	SGPA	Problematic mobile phone use scale
Uzun and Kilis (2019)	631	-0.107	Both	18–24	TE	CA	PPS	SGPA	Media and technology use and attitude scale
Wentworth and Middleton (2014)	483	0.01	Both	18–29	TE	LRA	PPS	SGPA	Total phone use (minutes/week)
Winskel et al. (2019)	119	0.04	Both	18–26	TE	CA	OS	SGPA	Total phone use (minutes/day).
Winskel et al. (2019)	270	-0.3	Both	18–26	TE	CA	OS	SGPA	Total phone use (minutes/day).
Han et al., (2019)	2,491	0.125	Both	18–26	TE	PA	OS	APS	Smartphone Self-Efficacy scale
Bai et al. (2020)	1,794	0.128	Both	7–18	P/S	SEM	CS	GPA	Mobile Phone Addiction Index
Coskun and Karayagız Muslu (2019)	1,603	-0.28	Both	15–18	S	RA	CS	GPA	Problematic Mobile Phone Use Questionnaire
Domoff et al. (2020)	641	0.170	Both	15–18	S	CA	CS	SRG	Addictive Patterns of Use Scale
Eoh et al. (2022)	695	-0.18	Both	10–11	P/S	MMA/ LRA	CS	TS	Internet Addiction Diagnostic Scale
Przepiorka et al. (2021);	209	-0.2	Male	10–14	P/S	CA	CS	GPA	Smartphone Addiction Scale-Short Version
Przepiorka et al. (2021)	218	-0.26	Female	10–14	P/S	CA	CS	GPA	Smartphone Addiction Scale-Short Version
Wu and Siu (2020)	411	-0.134	Both	15–18	S	CA/LRA	CS	SRG	Problematic Cellular Phone Use Questionnaire
Zhou et al. (2022)	19,845	-0.21	Both	10	P	CA	OS	TS	Problematic smartphone use
Social media use and academic performance									
Shafiq and Parveen (2023)	234	0.142	Both	18–24	TE	SEM	S	AGPA	Time used on SM
Homaid (2022)	312	0.552	Both	18–27	TE	SEM	OS	APDS	Time spent on social media
Dubuc et al. (2020)	187	-0.02	Both	12–14	P	PPC	L	AGPA	SM usage frequency
Tomé-Fernández et al. (2020)	624	0	Both	8–17	P/S	DSA	CS	SGPA	Communication and social interaction on SM
Bergdahl et al. (2020)	410	-0.19	Both	15–18	S	BCA	CS	SGPA	Time used on SM
Wakefield and Frawley (2020)	505	-0.067	Both	15–18	S	RA	CS	FEP	Time spent in total on facebook
Alotaibi (2019)	395	-0.181	Both	15–18	S	MLR	S	SGPA.	SM activity
Bardakcı (2019)	335	0.8	Both	15–18	S	SEM	S	SGPA	Using YouTube for educational purpose
Sampasa-Kanyinga et al. (2019)	10,076	-0.153	Both	12–18	S	MLR	S	SGPA	Number of hours spend on SM
Van Den Eijnden et al. (2018)	538	-0.33	Both	12–15	S	DS	OS	ASG	SM use
Tang and Patrick (2018)	40,389	0.03	Both	14–16	S	LR	S	ASG	Time used on SM
Arora et al. (2018)	853	-0.07	Both	12–15	S	MA	S	AR	Time used on SM
Çimen and Yılmaz (2017)	104	0.463	Both	16	S	PPE	PPT	AATS	Log-in times intensity of sharing
Yan and Yang (2017)	2,625	-0.178	Both	15–18	S	LLR	S	SGPA	Time used on SM
Qahri-Saremi and Turel (2016)	6,885	-0.03	Both	14–16	S	A/SEM	S	SGPA	Time used on SM
Tsai and Liu (2015)	1,365	-0.407	Both	13–15	S	MR	S	SGPA	A scale of Students’ FB interpersonal skills
Tsitsika et al. (2014)	10,930	-0.12	Both	14–17	S	DS	OS	SGPA	Time used on SM
Khan et al. (2014)	690	0.09	Both	15–18	S	PC	PPS	SGPA	FB visit frequency
Videogames use and academic performance									
Brunborg et al. (2014)	1928	-0.17	Both	13–17	S	RA	L	SG	Game Addiction Scale for Adolescents
Schmitt and Livingston (2015)	477	-0.12	Female	18	TE	CA	L	GPA	Self-developed videogame addiction scale
Zorbaz et al. (2015)	396	-0.271	Both	10–11	P	CA	CS	GPA	Scale Of Game Addiction for Children
Sahin et al. (2016)	370	-0.222	Both	-	S	CA	CS	GPA	Game Addiction Scale
Hawi et al. (2018)	524	-0.183	Both	15–19	S	MRA	CS	GPA	Internet Gaming Disorder Test
ELNahas et al. (2018)	996	0	Both	18-24	TE	CA	CS	AG	Internet Gaming Disorder Scale
Shi et al. (2019)	1,275	-0.1	Both	7–12	P	CA	CS	SG	Problem Video Game Playing Scale
Samaha and Hawi (2020)	345	-0.179	Both	18–24	TE	CA	CS	GPA	Internet Gaming Disorder Test
Zahra et al. (2020)	315	-0.23	Both	18–25	TE	CA	CS	AG	Internet Gaming Disorder Test
Ekşi et al. (2020)	206	-0.065	Both	14–18	S	SEM	CS	GPA	Digital Game Addiction Scale
Jaafar et al. (2021)	411	-0.18	Both	19–25	TE	CA	CS	GPA	The Internet Gaming Disorder Scale-Short-Form
Karnadi and Pangestu (2021)	390	-0.056	Both	19–25	TE	BD	CS	GPA	Internet Gaming Disorder Test
Suryawanshi et al. (2021)	91	-0.02	Both	19–25	TE	CA	CCD	AG	The Gaming Addiction Scale
Ciris et al. (2022)	559	0.108	Both	15–18	S	CA	CS	GPA	Game Addiction Scale for Adolescents
Polat and Topal (2022)	289	-0.259	Both	12–13	S	CA	CS	GPA	Digital Game Addiction Scale
Yang et al. (2022)	195	-0.17	Both	18–22	TE	CA	L	GPA	Young’s Internet Addiction Test
Total Research: 63 Total Sample Size: 124.166									

Grade Level: TE, tertiary education; S, secondary education; P, primary education. Empirical Design: LRA, logistic regression analysis; PA, path analysis; CA, correlational analysis; SEM, structural equation model; RA, regression analysis; MMA/ LRA, moderated mediation analysis/logistic regression analysis; PPC, Pearson’s partial correlation; DS/A, descriptive statistics/ANOVA; BC/A, bivariate correlation/ANOVA; MLR, multiple linear regression; DS, descriptive statistic; LR, logistic regression; MA, mediation analysis; PPE, pre-post experimental; LLR, linear and logistic regression; A/SEM, ANCOVA/SEM; MR, multiple regression; PC, Pearson correlation; BD, binary dependent and two-staged least squares models. Research Design: OS, online survey; PPS, paper pen survey; CS, cross sectional; S, survey; L, longitudinal; PPT, pre-and post-test; CCD, case–control design. Academic Performance Indicator: AAS, academic achievement subscale; AGPA, actual GPA; GPA, grade point average; CAS, college adjustment scale; SGPA, self-reported GPA; APS, academic performance scale; SSCG, social science course grade; SRG, school report grades; TS, test scores; APDS, academic performance decrement scale; FEP, final examination performance; ASG, average school grades.

### 4.2 Results of meta-analysis: RQ1: what relationships exist between SA, SMU, VGs, and AP?

The meta-analysis results purported that SA and VGs have a small negative impact on AP, whereas SMU’s impact is ambivalent. Table 3 illustrates the result of the meta-analysis.

**Table 3:** Meta-analysis results for SA, SMU, VGs.

Academic performance	Sample		Effect size statistic				Heterogeneity				Publication Bias
Subgroups	*k*	*N*	Estimate *(d)*	se	*p*	%95 CI	Tau^2^	*I* ^2^	*Q*	*p*	Begg and Mazumdar p
Smartphone	29	37,942	−0.129	0.027	<0.001	[−0.183 – −0.075]	0.018	%94.76	671.191	<0.001	0.368
Social Media	18	77,457	0.025	0.038	>0.514	[−0.051–0.102]	0.025	%98.73	1342.301	<0.001	0.081
Video Games	16	8,767	−0.134	0.027	<0.001	[−0.187 – −0.082]	0.008	%82.27	81.257	<0.001	0.825
Total	63	124.166	−0.085	0.029	<0.004	[−0.143 – −0.028]	0.051	%98.86	2501.926	<0.001	0.400

**Figure 2:**
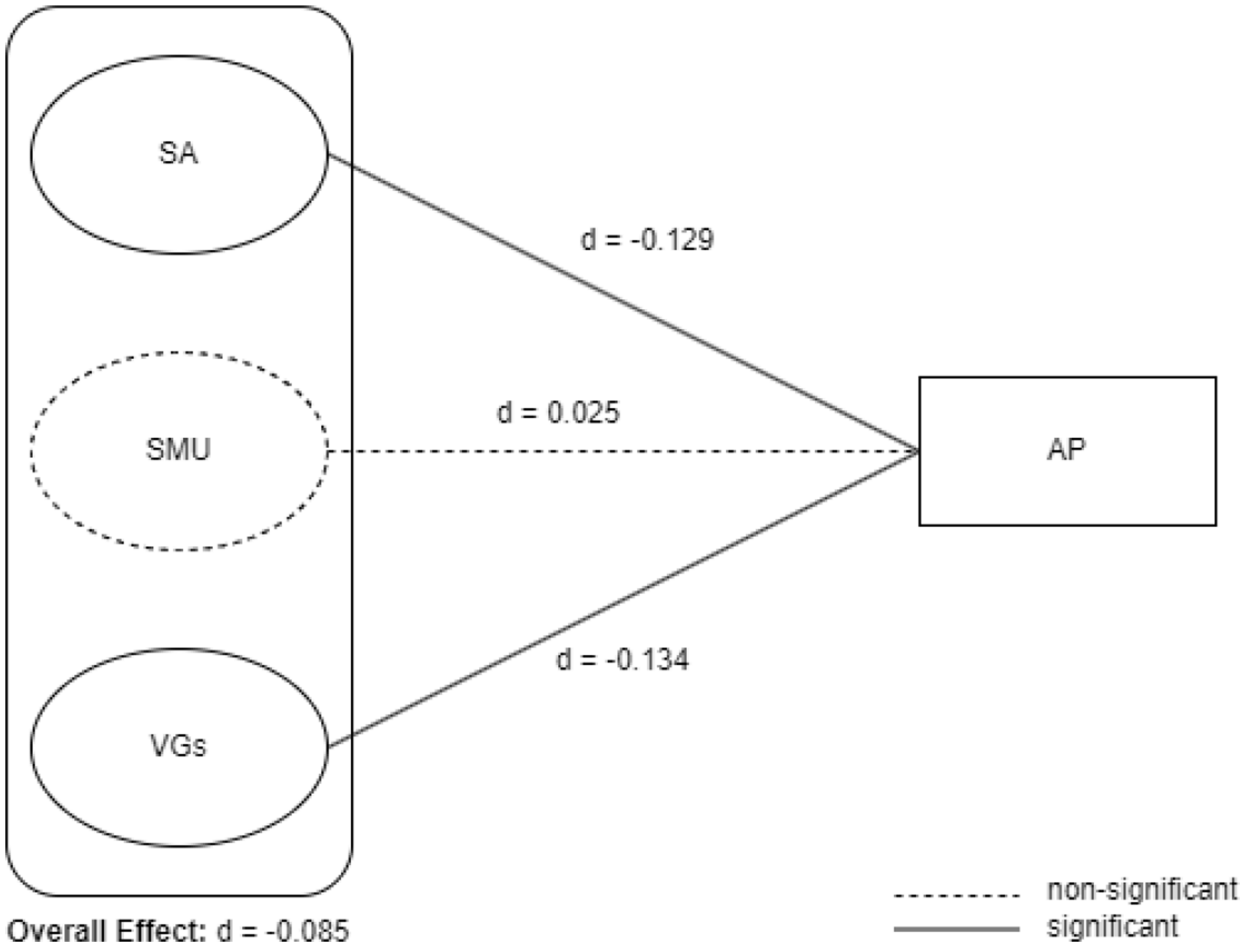
Hypothesized model of meta-analysis.

**Figure 3:**
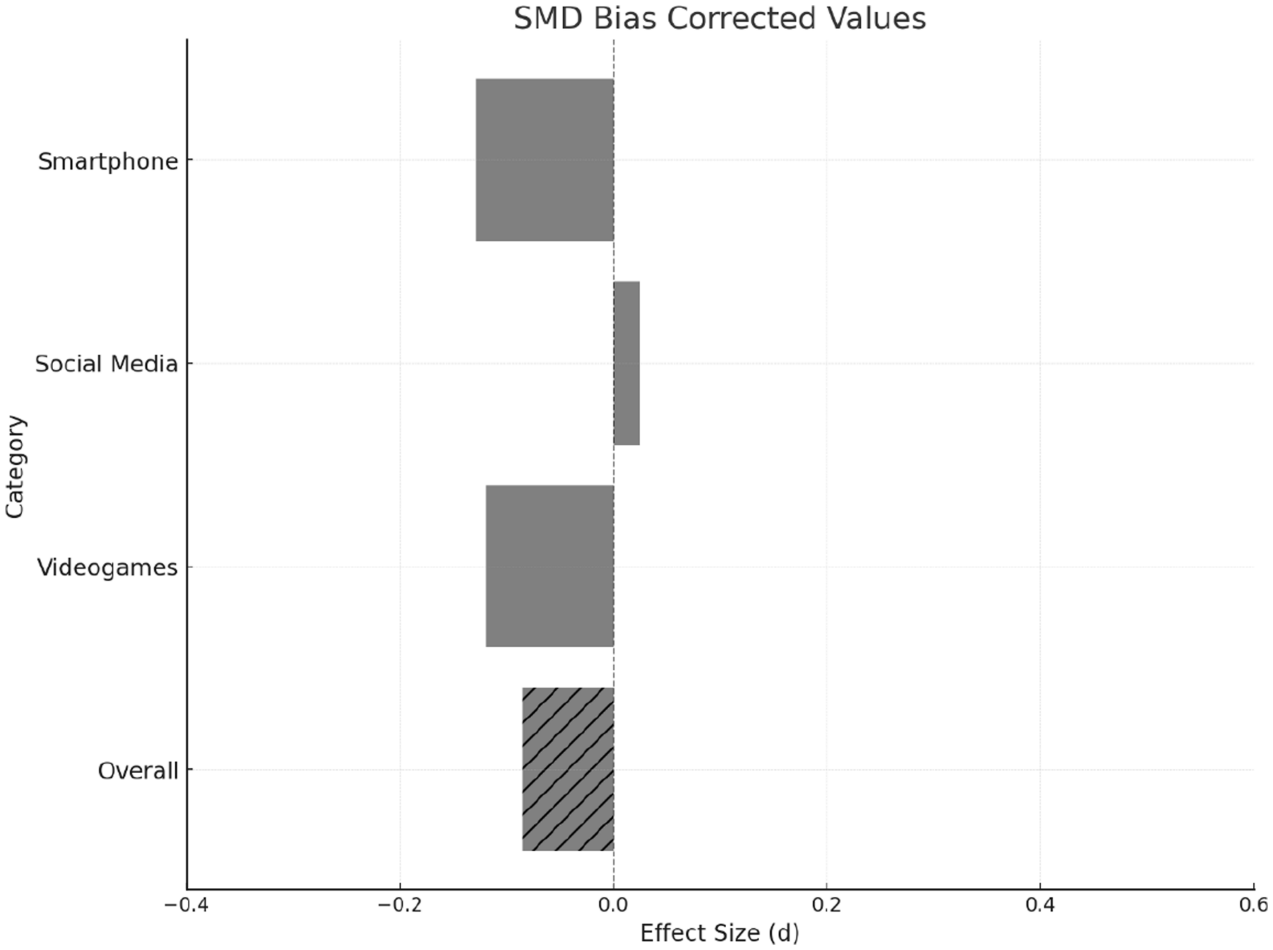
SMD bias corrected values by variables.

According to the results of the meta-analysis, the effect sizes on academic performance are as follows: for SA, *d* = −0.129, 95% CI [−0.183, −0.075], indicating a small negative effect; for SMU, *d* = 0.025, 95% CI [−0.051, 0.102], showing a small positive effect, although this effect is not statistically significant. For VGs, *d* = −0.134, 95% CI [−0.187, −0.082], there is a small negative effect. The overall effect of all factors on AP is *d* = −0.085, 95% CI [−0.143, −0.028], indicating a small negative effect (Figures 2, 3).

### 4.3 Publication bias of subgroup analysis

According to the Begg and Mazumdar test, *p* > 0.394, suggesting no evidence of publication bias. The forest plot and funnel plot for the results are presented in the following figures, respectively.

**Figure 4:**
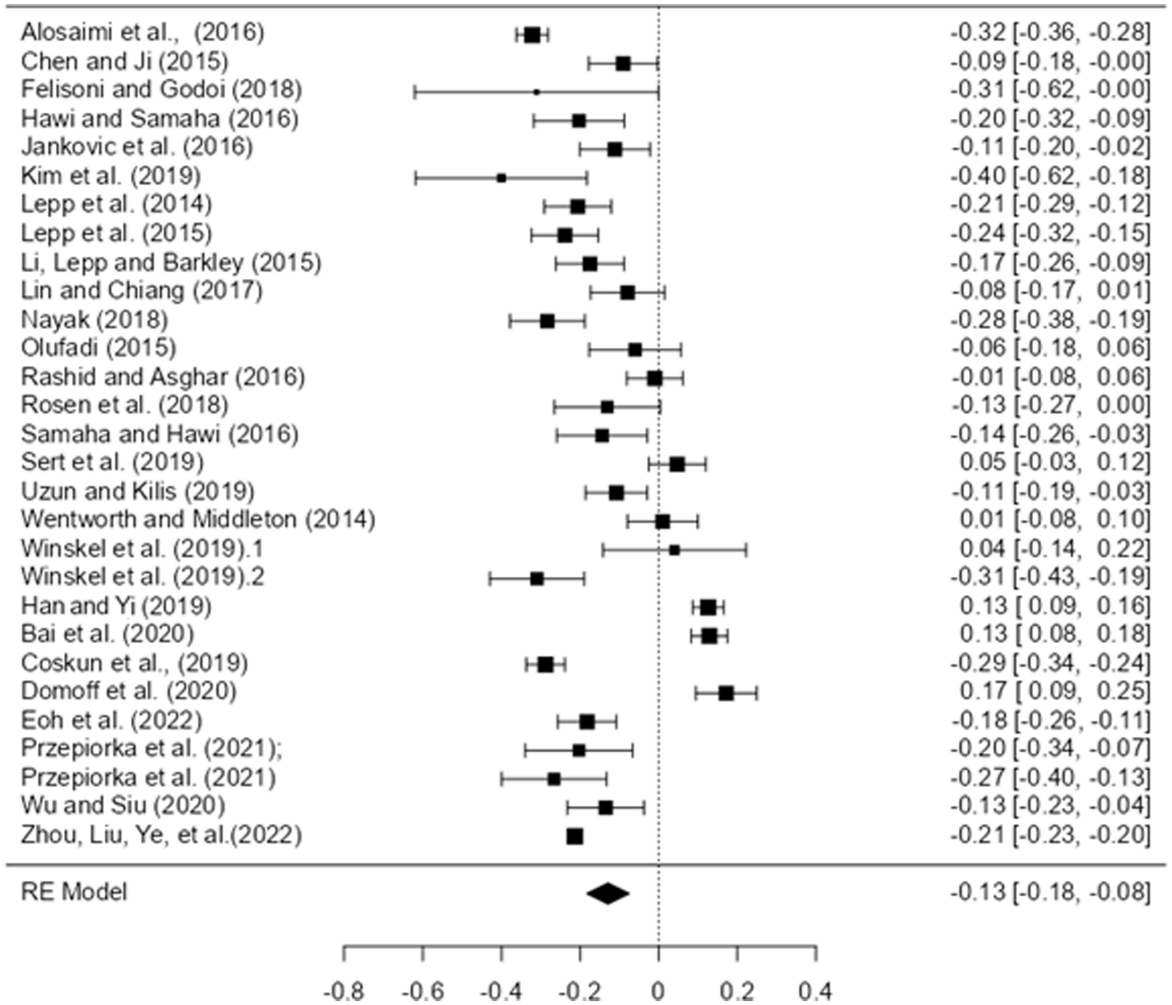
Forest plot of smartphone results.

Figure 4 presents a forest plot showing the combined effect sizes obtained from the smartphone variable.

**Figure 5:**
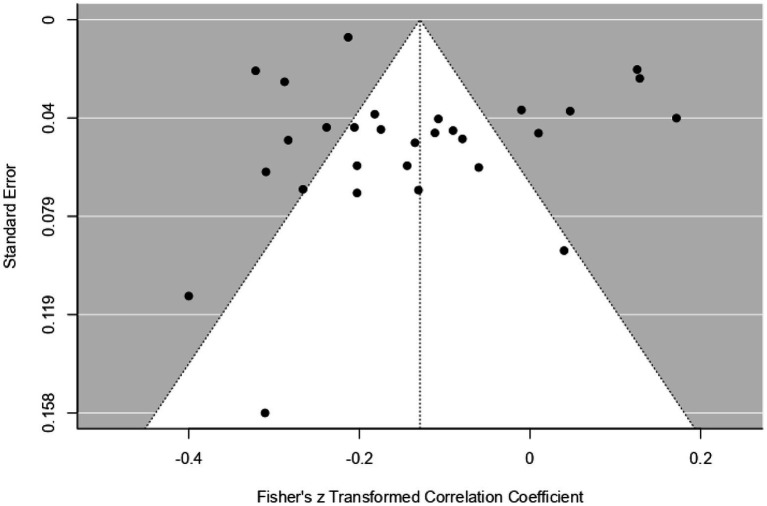
Funnel plot of smartphone results.

Figure 5 presents a funnel plot assessing the distribution of data obtained from the smartphone.

**Figure 6:**
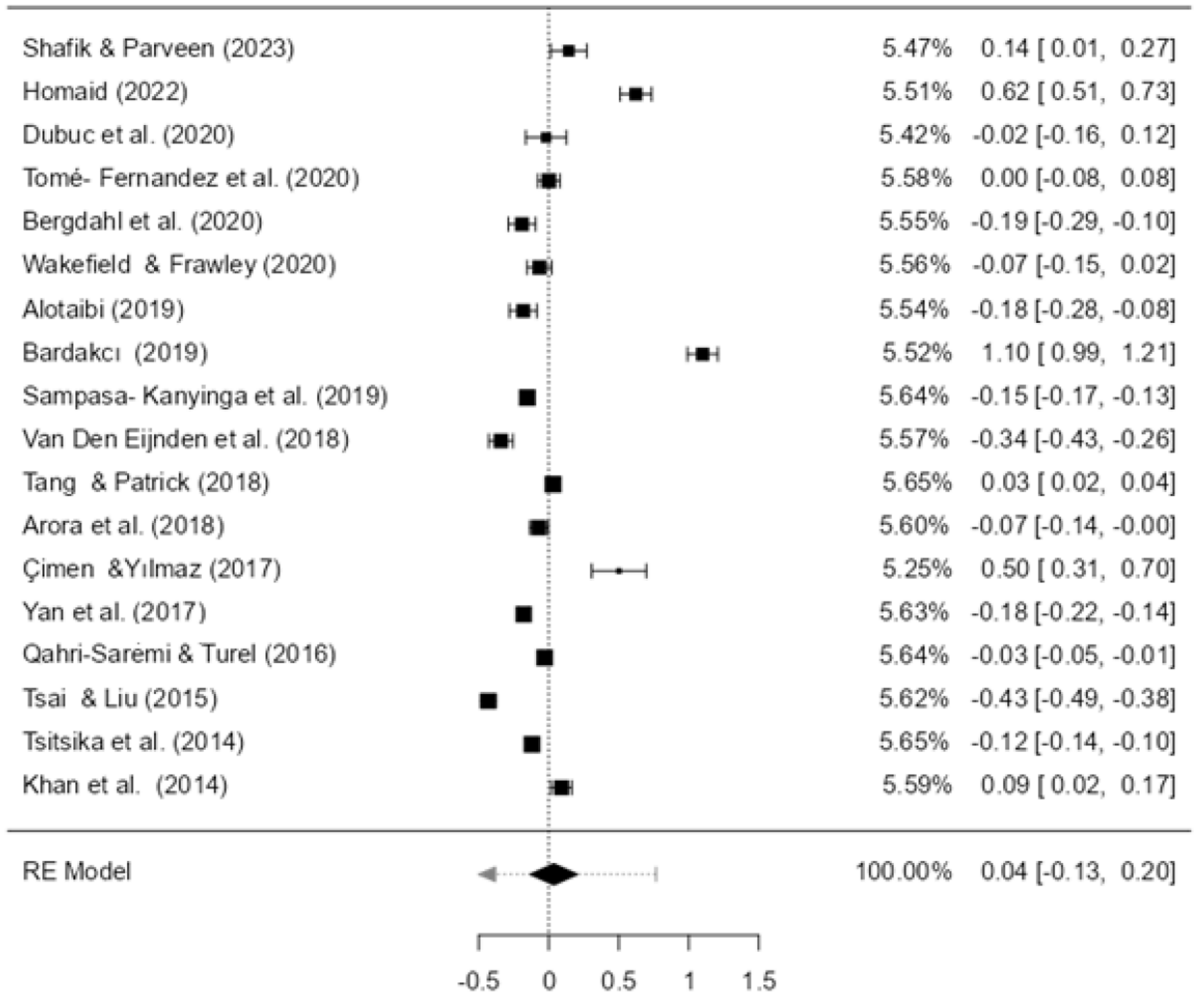
Forest plot of social media results.

Figure 6 presents a forest plot showing the combined effect sizes obtained from the social media variable.

**Figure 7:**
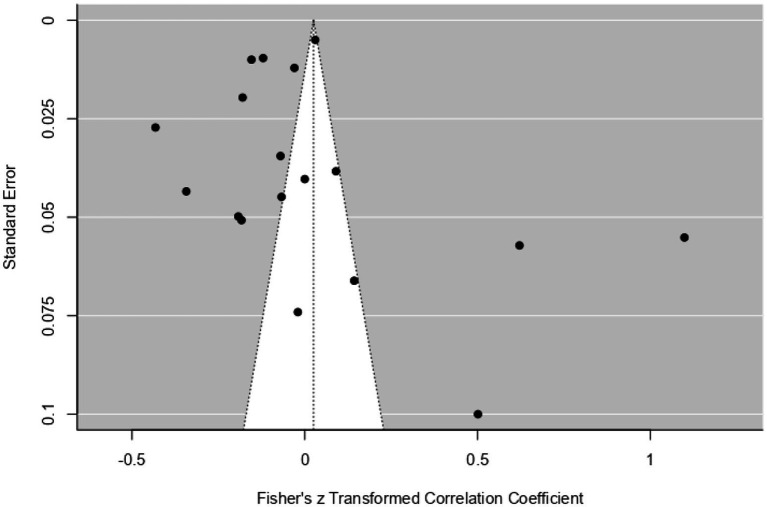
Funnel plot of social media results.

Figure 7 presents a funnel plot assessing the distribution of data obtained from the social media.

**Figure 8:**
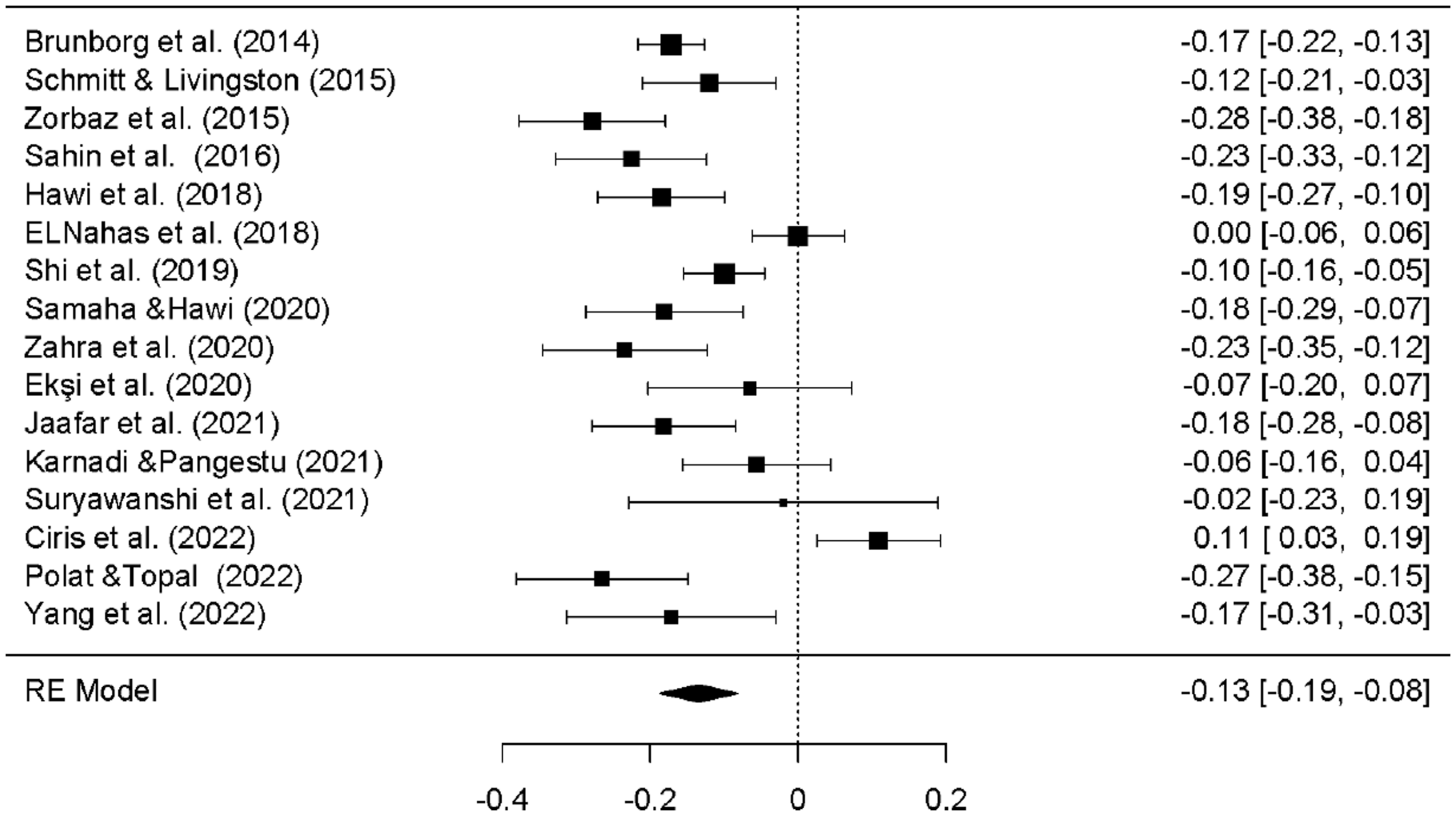
Forest plot of videogames results.

Figure 8 presents a forest plot showing the combined effect sizes obtained from the video games variable.

**Figure 9:**
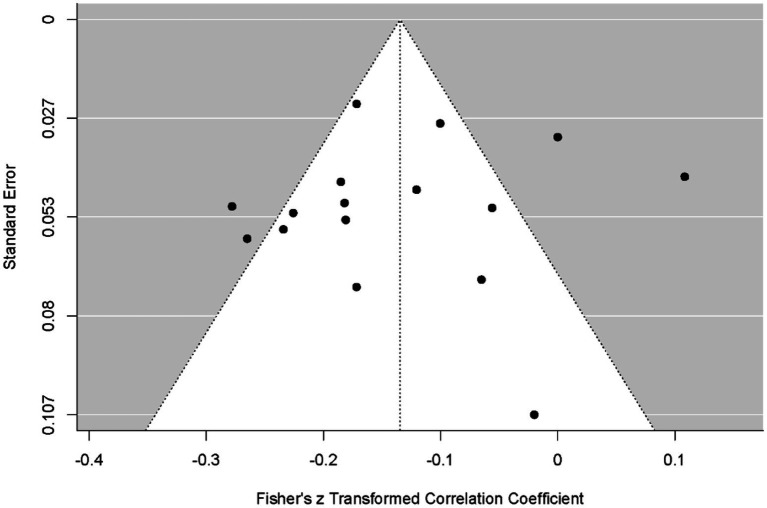
Funnel plot of videogames results.

Figure 9 presents a funnel plot assessing the distribution of data obtained from the video games.

**Figure 10:**
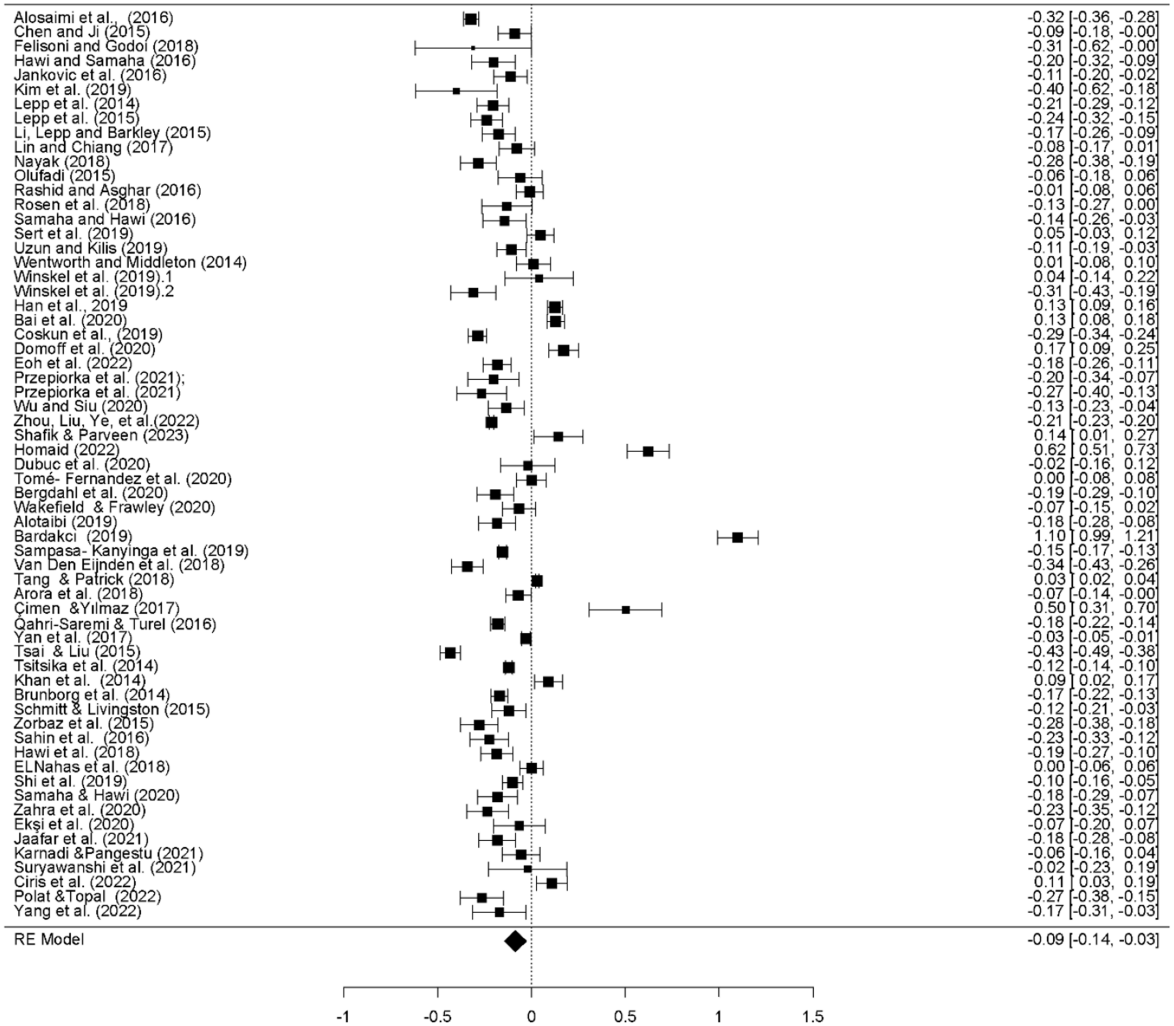
Forest plot of combined results for all factors.

Figure 10 presents a forest plot displaying the combined effect sizes obtained from the results of all factors.

**Figure 11:**
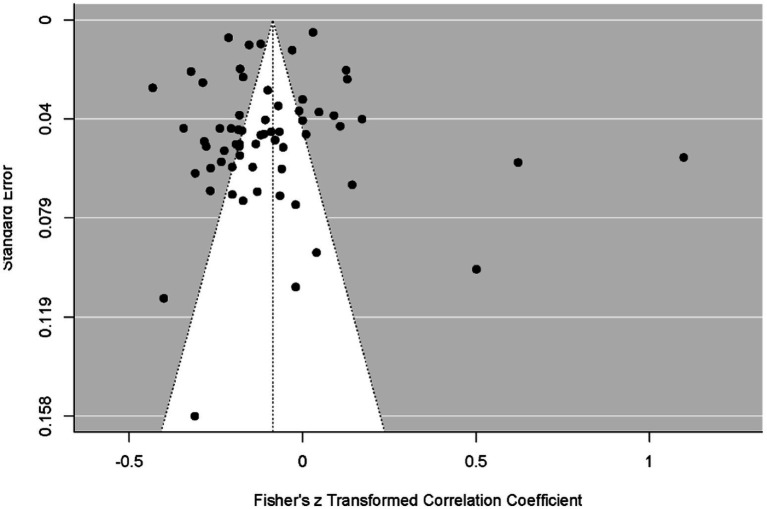
Funnel plot of combined results for all factors.

Figure 11 presents a funnel plot assessing the distribution of data obtained from all factors.

**Figure 12:**
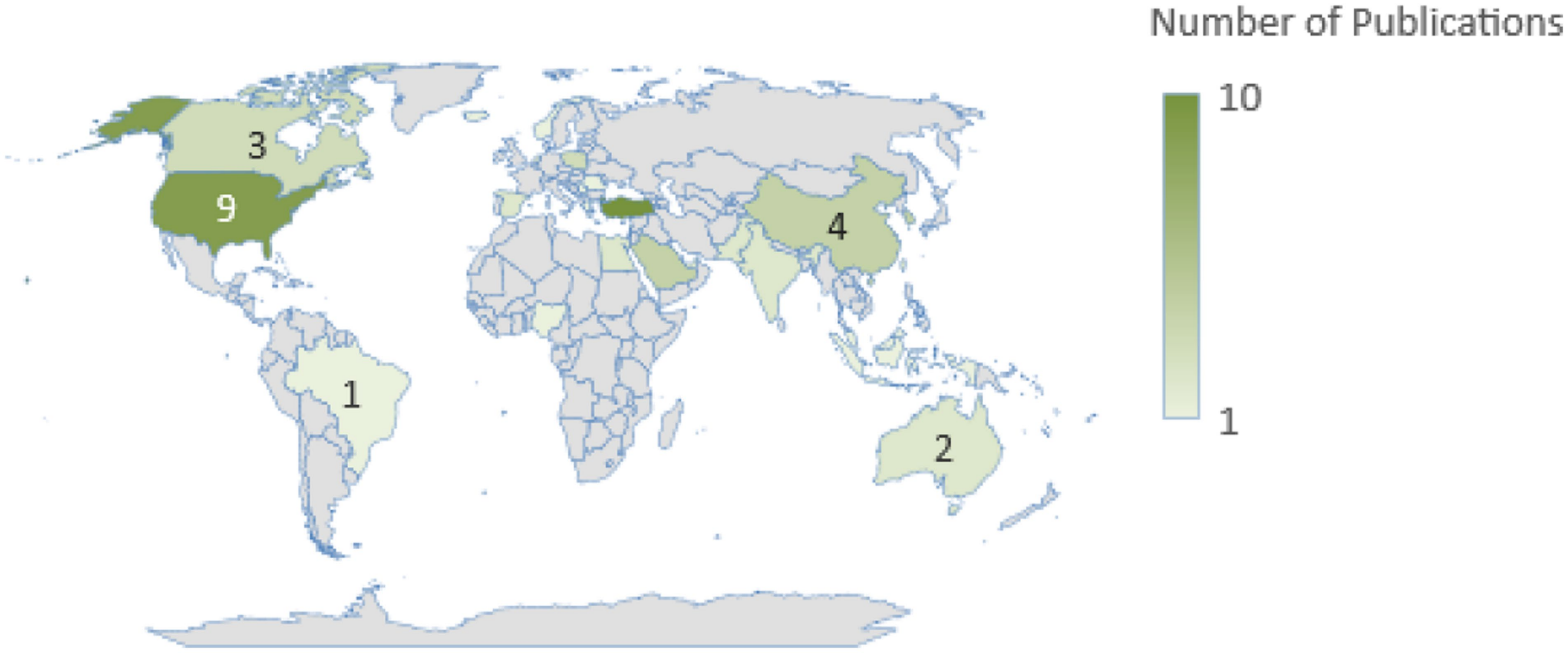
Distribution of the reviewed publications on the world map.

Additionally, the countries where the studies were conducted are presented as Supplementary material, and a heat map has been created on a world map to highlight the countries with the highest study count. The heat map is shown in Figure 12.

According to the meta-analysis results, Turkey has the highest number of publications (10), followed by the following countries: United States (9), Lebanon (4), South Korea (4), China (4), Saudi Arabia (4), Poland (3), Canada (3), Egypt (1), United Arab Emirates (2), Taiwan (2), Spain (2), Pakistan (2), India (2), Australia (2), Netherlands (2), Romania (1), Indonesia (1), Malaysia (1), Brazil (1), Norway (1), Iceland (1), Serbia (1), Greece (1), Singapore (1), Sweden (1), Nigeria (1), Hong Kong (1).

### 4.4 Moderation analysis results: RQ2: what factors moderate the relationship between SA, SMU, VGs, and AP as demonstrated by past research?

As a secondary analysis, as subgroup analysis was conducted in order to investigate that age and educational level variables significantly moderate the relationship between SA, SMU, VGs, and AP. The results were presented in Table 4.

**Table 4:** Meta-analysis results on the impact of moderator variables in the relationship between factors and academic performance.

Academic performance		Sample		Effect size statistic				Heterogeneity	
	Moderator variables	*k*	*N*	Estimate *(d)*	se	*p*	%95 CI	*I* ^2^	*Q*
Smartphone	Intercept	29	37,942	−0.106	0.108	0.327	[0.320–0.107]	%93.80	623.599
	Age			−0.013	0.061	0.832	[−0.121–0.072]		
	Intercept	29	37,942	−0.131	0.050	0.009	[0.230 – −0.033]	%94.01	654.528
	Grade level			−0.001	0.026	0.950	[−0.049–0.053]		
Social media	Intercept	18	77,547	0.506	0.432	0.241	[−0.341–1.353]	%99.76	1257.834
	Age			−0.256	0.231	0.266	[−0.709–0.196]		
	Intercept	18	77,457	0.453	0.343	0.187	[−0.219–1.125]	%99.76	1268.187
	Grade level			−0.001	0.026	0.950	[−0.384–0.084]		
Video games	Intercept	16	8,767	−0.100	0.094	0.283	[−0.285–0.083]	%82.88	78.074
	Age			−0.021	0.056	0.707	[−0.132–0.089]		
	Intercept	16	8,767	−0.122	0.064	0.056	[−0.248 – −0.003]	%82.77	78.757
	Grade level			−0.006	0.030	0.830	[−0.066–0.053]		
Total factor	Intercept	63	124.166	−0.110	0.063	0.079	[−0.234 – −0.013]	%98.86	2489.315
	Age			0.012	0.027	0.651	[−0.042–0.067]		
	Intercept	63	124.166	−0.026	0.116	0.823	[−0.253–0.201]	%98.71	2238.449
	Grade level			−0.034	0.065	0.596	[−0.163–0.093]		

In Table 4, for values exhibiting high heterogeneity (*I*^2^ > 25%), the moderating effects of factors considered to influence AP were examined, with particular attention to age and educational level. The analyses indicated that the assessed moderator variables did not have a statistically significant effect on AP.

## 5 Discussion

This meta-analysis provides an in-depth investigation into the relationships among smartphone addiction (SA), social media use (SMU), video games (VGs), and academic performance (AP) across primary, secondary, and tertiary education levels. While smartphones and digital technologies are increasingly integrated into students’ lives, there remains a significant gap in the literature concerning their potential as learning tools and their impact on AP. By synthesizing data from 63 studies encompassing 124,166 participants, this analysis offers valuable insights for education policymakers and practitioners regarding the implications of SA, SMU, and VGs on students’ academic outcomes. The findings of this study shed light on the degree to which SA, SMU, and VGs influence academic achievement, and whether these effects are moderated by variables such as age, and educational level.

First, the results demonstrate a weak but statistically significant negative association between SA and AP (d = −0.129, 95% CI [−0.183, −0.075]). **Hypothesis 1 (SA → AP):** Accepted (statistically significant negative effect). supporting prior research indicating that excessive smartphone use can detract from students’ academic success. Several studies have similarly reported negative correlations between SA and AP, suggesting that smartphone overuse may divert attention away from academic responsibilities, leading to poorer academic outcomes (Asante and Hiadzi, 2018; Durak, 2018; Felisoni and Godoi, 2018; Li et al., 2015; Longnecker, 2017; Mendoza et al., 2018; Olufadi, 2015; Rozgonjuk et al., 2018; Spiratos, 2021; Zhou et al., 2022).

Second, the effect of SMU on AP was found to be weakly positive, though not statistically significant (d = 0.025, 95% CI [−0.051, 0.102]). **Hypothesis 2 (SMU → AP):** Rejected (not statistically significant). This suggests that while social media use may not substantially enhance academic performance, it does not appear to exert a strong detrimental effect either. This aligns with other studies that highlight the potential of social media as a resource for academic collaboration, information sharing, and peer learning (Dubuc et al., 2020; Kalam et al., 2023; Lim et al., 2021; Tomé-Fernández et al., 2020).

Third, the analysis revealed a weak negative association between VGs and AP (*d* = −0.134, 95% CI [−0.187, −0.082]). **Hypothesis 3 (VG → AP):** Accepted (statistically significant negative effect). This finding is consistent with the literature, which indicates that excessive gaming may hinder academic performance due to its time-consuming nature and its potential to displace more productive academic activities (Brunborg et al., 2014; Ekşi et al., 2020; Ferguson, 2015; Samaha and Hawi, 2020; Schmitt and Livingston, 2015; Shi et al., 2019; Suryawanshi et al., 2021; Toker and Baturay, 2016; Zhang et al., 2019; Zorbaz et al., 2015). However, the overall effect of SA, SMU, and VGs on AP (d = −0.085, 95% CI [−0.143, −0.028]). **Hypothesis 4 (SA, SMU, VG → AP):** Accepted (statistically significant combined negative effect). This finding suggests that, when considered together, these digital behaviors exert only a weak negative influence on academic outcomes. Contrary to widespread concerns about the harmful effects of digital technology, these findings imply that, under certain conditions, SA, SMU, and VGs may not be as damaging to academic success as is commonly believed. Moreover, the nuanced results highlight the complex interplay between these factors and AP, suggesting that their impact may vary based on individual usage patterns and contexts.

When examining potential moderators of the relationship between SA, SMU, VGs, and AP, variables such as gender, age, and educational level were analyzed. The findings revealed no significant moderating effect of these variables on academic performance, suggesting that the influence of SA, SMU, and VGs on AP is relatively consistent across different demographic groups. This result underscores the importance of further exploring other potential moderating factors, such as cultural context, socioeconomic status, or psychological traits, which may help explain variations in how these digital behaviors impact AP.

## 6 Conclusion

The findings of this meta-analysis contribute to a nuanced understanding of the relationships among smartphone addiction (SA), social media use (SMU), video games use (VGs), and academic performance (AP). The results indicate that both SA and VGs usage exert a weak negative impact on students’ academic performance, while SMU demonstrates a weak, albeit statistically insignificant, positive effect. Excessive engagement with smartphones, social media, and video games—particularly when it interferes with daily responsibilities—can lead to behavioral addiction, which adversely affects academic outcomes.

Consequently, fostering self-control and encouraging students to moderate their smartphone use may yield beneficial effects on their academic performance. Based on the findings of this study, we advocate for the implementation of educational policies, targeted teacher training, cognitive-behavioral interventions, and the development of specific teaching and learning strategies that prioritize harm reduction. These measures should address the potential for addiction associated with smartphone use, social media, and video games. Such initiatives could facilitate optimal study habits and enhance students’ academic performance.

This meta-analysis highlights several avenues for future research while acknowledging inherent limitations. Longitudinal studies are essential to explore the long-term impacts of smartphone addiction, social media use, and video game engagement on academic performance, thereby facilitating a clearer understanding of causal relationships over time. Additionally, research should extend to diverse populations, encompassing various age groups, cultural backgrounds, and educational systems to enhance the generalizability of findings. Employing qualitative methodologies could further enrich insights into students’ experiences and perceptions regarding digital behaviors and their academic consequences. Moreover, intervention studies aimed at reducing problematic usage patterns while promoting beneficial aspects of social media for academic enhancement are warranted. Future investigations should also consider additional moderating factors, such as socioeconomic status, personality traits, and contextual influences, that may impact the relationships between smartphone addiction, social media use, video game engagement, and academic performance.

Despite these potential directions, this meta-analysis is not without its limitations. The heterogeneity of the included studies, which varied in methodologies, measures of academic performance, and definitions of the key constructs, may affect the consistency and comparability of results. This meta-analysis is not without its limitations. One of the primary challenges lies in the conceptualization and measurement of SA, SMU, and VGs. Given the pervasive and multifaceted nature of smartphone use, it is difficult to distinguish between casual, problematic, and addictive use, which may lead to inconsistent findings across studies. There is a pressing need for the development of more robust and standardized tools to accurately assess these behaviors. Furthermore, the emerging nature of SA, SMU, and VGs, coupled with their rapid socio-cultural integration, has limited the availability of high-quality research on their academic effects. This limitation may have constrained the scope of this meta-analysis. Additionally, the possibility of publication bias remains, despite efforts to incorporate both published and unpublished studies, potentially influencing the findings. Moreover, while the overall sample size is substantial, many individual studies had small sample sizes or narrow demographic ranges, which limits the ability to draw broad conclusions. Furthermore, reliance on self-reported data for measuring academic performance and usage behaviors can introduce biases that may affect the accuracy of the findings. Lastly, the predominance of cross-sectional designs in the included studies restricts the capacity to establish definitive causal relationships among the variables examined.

## Data Availability

The original contributions presented in the study are included in the article/Supplementary material, further inquiries can be directed to the corresponding author/s.
